# Mass Spectrometry Imaging of *Arabidopsis thaliana* Leaves at the Single-Cell Level by Infrared Laser Ablation Atmospheric Pressure Photoionization (LAAPPI)

**DOI:** 10.1021/jasms.1c00295

**Published:** 2021-11-05

**Authors:** Juha-Pekka Hieta, Nina Sipari, Heikki Räikkönen, Markku Keinänen, Risto Kostiainen

**Affiliations:** † Drug Research Program and Division of Pharmaceutical Chemistry and Technology, Faculty of Pharmacy, University of Helsinki, P.O. Box 56, Helsinki 00014, Finland; ‡ Viikki Metabolomics Unit, Faculty of Biological and Environmental Sciences, University of Helsinki, P.O. Box 56, Helsinki 00014, Finland; § Department of Environmental and Biological Sciences, Institute of Photonics, Faculty of Science and Forestry, University of Eastern Finland, P.O. Box 111, Joensuu 80101, Finland

## Abstract

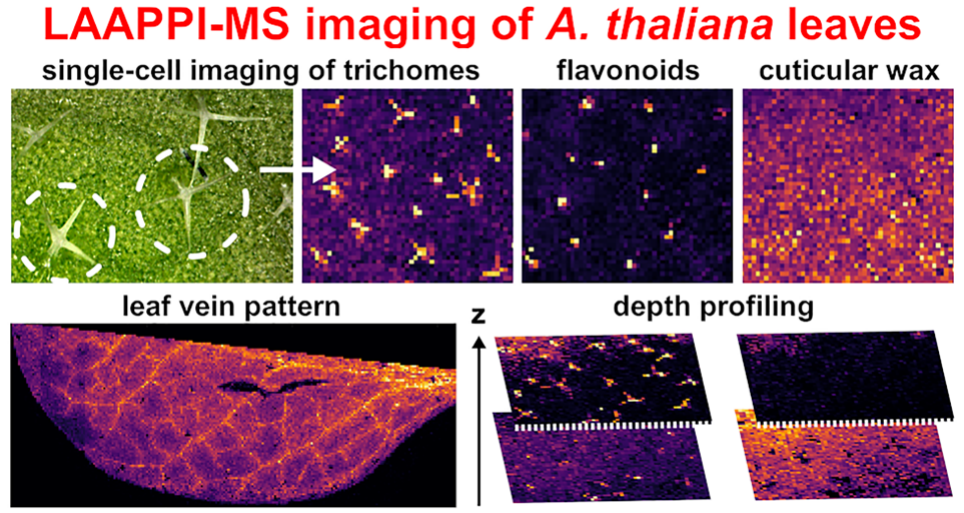

In this study, we show that infrared laser ablation atmospheric pressure photoionization mass spectrometry (LAAPPI-MS) imaging with 70 μm lateral resolution allows for the analysis of *Arabidopsis thaliana* (*A. thaliana*) leaf substructures ranging from single-cell trichomes and the interveinal leaf lamina to primary, secondary, and tertiary veins. The method also showed its potential for depth profiling analysis for the first time by mapping analytes at the different depths of the leaf and spatially resolving the topmost trichomes and cuticular wax layer from the underlying tissues. Negative ion LAAPPI-MS detected many different flavonol glycosides, fatty acids, fatty acid esters, galactolipids, and glycosphingolipids, whose distributions varied significantly between the different substructures of *A. thaliana* leaves. The results show that LAAPPI-MS provides a highly promising new tool to study the role of metabolites in plants.

## Introduction

Various mass spectrometry (MS) methods have recently been developed and employed for single-cell analysis.^1−3^ Among these, vacuum-based ultraviolet matrix-assisted laser/desorption ionization (UV-MALDI)^4^ and secondary ion mass spectrometry (SIMS)^5^ are often used for MS profiling and imaging of single cells because they provide fast analysis, high sensitivity, and high lateral resolution. However, SIMS suffers from extensive analyte fragmentation^6^ that complicates identification, and the mandatory matrix deposition step in MALDI results in an increased chemical background and can cause a delocalization of analytes in the sample.^7^ Most ambient mass spectrometry^8^ methods offer a straightforward alternative to single-cell analysis, as the ionization and desorption of intact biomolecules can be carried out in an open-air environment without additional sample treatment steps, such as dehydration or matrix deposition. As such, ambient MS methods enable the measurements of live cells in their native microenvironment with minimal perturbation of the cultured cells or tissue.^9^

A widely utilized set of ambient MS methods is based on infrared (IR) laser ablation (LA),^10^ which can perform accurate, matrix-free spot-to-spot sampling for MS analysis with good repeatability as the IR laser energy is absorbed by the sample water. IRLA sampling results in efficient ablation of sample molecules to the gas phase, after which they are subsequently ionized with an ionization method of choice, such as atmospheric pressure photoionization (APPI)^11^ or electrospray ionization (ESI),^12^ enabling the analysis of a wide range of molecules. Due to the problems in focusing the OPO IR laser beams, the IRLA methods were previously inappropriate for MS imaging that could spatially resolve cellular details from tissue. However, we recently showed that IRLA sampling can now ablate 20–40 μm diameter spots by using simple optics.^13^ We also demonstrated the use of this focusing method for MS imaging of rodent brain tissue sections with a 70 μm lateral resolution, showing that IR laser ablation APPI-MS (LAAPPI-MS) and laser ablation ESI (LAESI-MS) can now reach a new quality level in imaging rodent tissue.^14^ Thus, far, LAAPPI-MS has not been used to measure the contents of a single cell from tissue, whereas LAESI-MS has already shown its potential for single-cell analysis, for example, in a recent study with *Allium cepa* tissue.^15^

Here, we apply the recently improved lateral resolution of LAAPPI-MS for MS imaging of leaves of the model organism *Arabidopsis thaliana* (*A. thaliana*), which is an important species in fundamental plant research.^16^*A. thaliana* leaves have a complex, multilayered structure including substructures such as trichomes (plant hair), cuticular and surface waxes, veins, and epidermis, which can be difficult to analyze and spatially resolve from each other with more traditional MS imaging methods, such as MALDI. The trichomes of *A. thaliana* leaves are star-shaped outgrowths of single cells of a few hundred micrometers in length.^17^ Trichomes protect plants from various biotic and abiotic stresses by varying their physical shape or chemical composition^18^ and shield against harmful UV radiation and excess light.^19^ The capability to analyze and image individual trichomes as well as other leaf substructures is important because their function, development, and biochemical composition are still not completely known. Previously, LAAPPI, LAESI, and UV-MALDI have been used in different leaf studies,^20−22^ but only LAESI and MALDI have examined *A. thaliana* leaves,^23−27^ and none of these methods have been applied to trichome imaging. In this study, we show the current capability of LAAPPI-MS for imaging single-cell trichomes and other substructures of untreated and frozen *A. thaliana* leaves. In addition, we demonstrate the potential of LAAPPI-MS for depth profiling analysis for the first time by showing its capability to spatially resolve the topmost trichomes and cuticle layer from the underlying parts of the leaf.

## Experimental Section

### Solvents

Milli-Q water (Merck Millipore, Molsheim, France) was used to adhere leaves onto microscope glass slides (see below), whereas toluene (CHROMASOLV Plus, Sigma-Aldrich, Steinheim, Germany) was used as a spray solvent in the negative ion LAAPPI-MS measurements.

### Plants and Tissue Samples

*Arabidopsis thaliana* wild-type Col-0 plants were grown in a growth chamber at 22 °C in a 3:1 mixture of soil and vermiculite under a 12/12 h light cycle (100 μmol photons m^–2^ s^–1^). Several intermediate-aged leaves^28^ from 4-week-old plants were subsequently collected and mounted at room temperature onto normal microscope glass slides adaxial (top) side up. A 0.1 mL droplet of Milli-Q water was pipetted on the surface of the slide, and a collected leaf was gently pressed against the droplet position with another glass slide, after which the sample slide was placed onto a block of dry ice, quenching the metabolite processes. The application of water and use of surface tension provided a convenient method to prepare flat leaf samples for imaging without the use of chemicals or extensive physical force that may cause rapid metabolic changes in plant tissue. All samples were stored at −80 °C before analysis, and they were analyzed without further sample treatment.

### Mass Spectrometry Imaging

**Figure 1:**
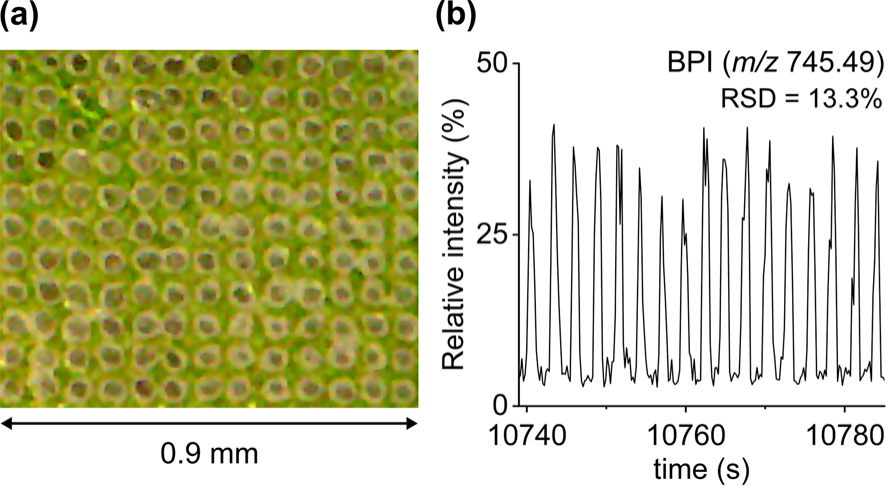
(a) Microscope image showing a grid of holes in the *A. thaliana* leaf lamina after MS imaging analysis with a 70 μm step size. (b) Repeatability of the negative ion LAAPPI-MS base peak ion trace (BPI, 34:6-MGD at *m*/*z* 745.49) measured from the *A. thaliana* leaf lamina (RSD = relative standard deviation).

LAAPPI-MS imaging was carried out with an in-house-built and enclosed dual LAAPPI/LAESI imaging platform that was previously described in detail.^14^ Briefly, the selected LAAPPI source consisted of a heated (350 °C) nebulizer for vaporizing the spray solvent (toluene, 1.0 μL min^–1^) and a VUV lamp (PKR 100; Heraeus Noblelight, Cambridge, UK) for initiating the photoionization with the emitted 10 eV photons, both pointed toward the MS inlet. The frozen leaf samples were mounted to a Peltier cooled (−24 °C) sample holder that was attached to X–Y–Z translation stages (NRT100/M; Thorlabs, Newton, MA) for rastering. The sublimation of ice onto the leaf surface was minimized by lowering the relative humidity within the enclosed imaging platform with a dry nitrogen flow. The MS inlet was equipped with a heated (200 °C) 24 mm long capillary extension to allow for spot-by-spot sampling of large sample areas with a nanosecond pulsed OPO IR laser (IR Opolette HE 2940; OPOTEK, Carlsbad, CA). The emitted IR photons ablated the analytes to gas phase, in which they encountered the ionized solvent and were ionized for MS analysis. The pulsed IR laser ablation sampling of 65 μm diameter spots perforating the whole leaf lamina (Figure 1) was accomplished by using simple optics^13^ and 20 consecutive laser pulses with 6 mJ output energy and 20 Hz repetition rate. LAAPPI-MS imaging analysis was carried out with 70 μm lateral resolution at a rate of 1500 pixels/h, and it was controlled with an in-house-developed MATLAB program. The mass spectra were acquired with a data acquisition frequency of 5 Hz in negative ion mode using a Bruker micrOTOF focus mass spectrometer (Bremen, Germany) and a mass resolution of ca. 10 000. The mass range was *m*/*z* 390–1000, covering the main secondary metabolites at the trichome regions of *A. thaliana* leaves. Level 5 identification^29^ was carried out for the main ions using their exact masses to match them with the most abundant compounds of *A. thaliana* leaves reported in the literature.

## Results and Discussion

### Negative Ion LAAPPI-MS Analysis of *A. thaliana* Leaves

**Figure 2:**
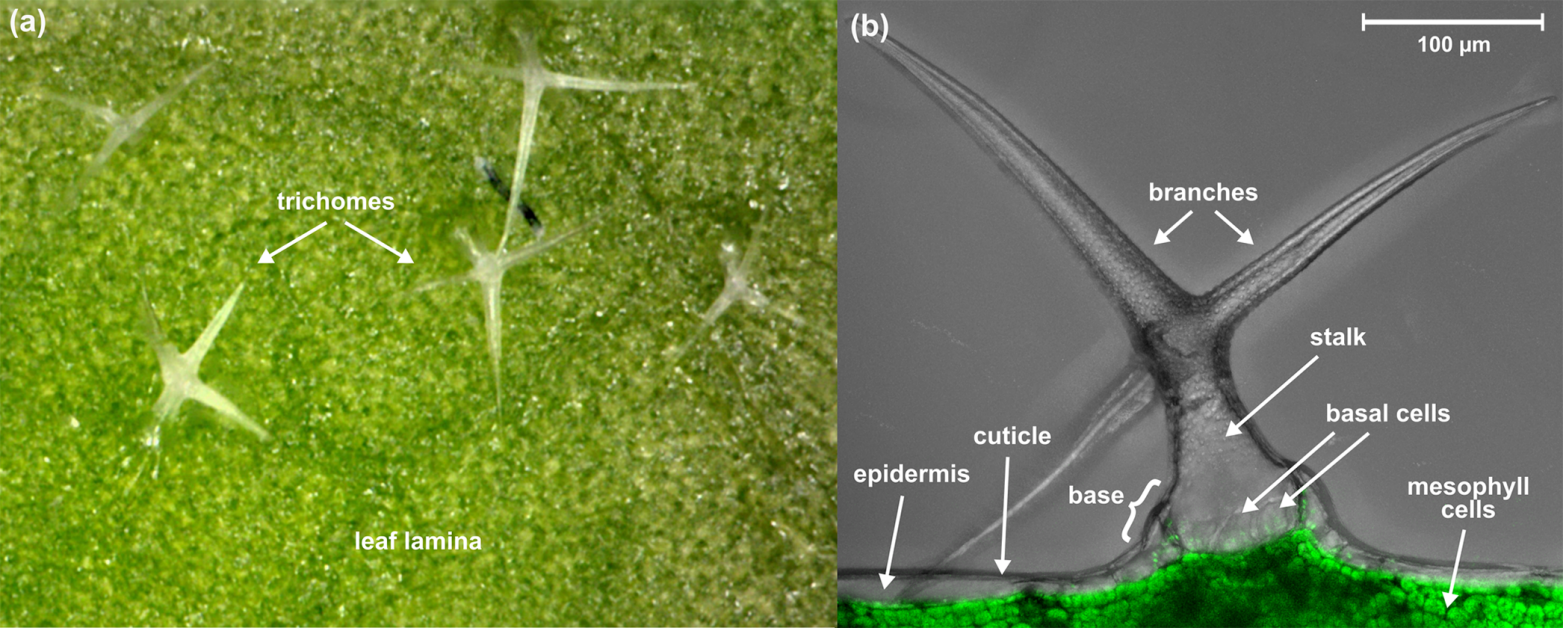
(a) Top view microscope image of the *A. thaliana* leaf surface showing multiple unicellular trichomes on the leaf lamina. (b) Side-view microscope image of the *A. thaliana* leaf surface showing a single-cell (unicellular) trichome (branches and stalk), basal cells, and the topmost leaf lamina. The image is combined with a fluorescence image that separates the chlorophyll-containing mesophyll cells from the epidermis.

The LAAPPI-MS sampling stability was assessed by calculating spot-to-spot repeatability using base peak intensity acquired from the interveinal leaf lamina tissue region (Figure 1a), in which the major leaf cell types, mesophyll and epidermal cells, are relatively uniformly distributed. The base peak ion trace (BPI, 34:6-MGD at *m*/*z* 745.49) showed a relative standard deviation (RSD) of 13.3% (Figure 1b), which indicates good spot-to-spot repeatability and stability of the method in imaging frozen *A. thaliana* leaves despite the highly uneven nature of the leaf surface (cf. tissue sections). This is consistent with our earlier study showing similar spot-to-spot repeatability (RSD = 16.8%) with frozen tissue sections of rodent brain.^14^ The sampling of more geometrically complex trichome regions was more inconsistent. The IR laser beam was focused on the leaf surface, whereas the trichomes rise hundreds of μm above it (Figure 2). The varying orientation of trichome branches relative to the IR laser beam is likely to the affect the sampling of underlying leaf parts and/or the direction of the resulting ablation plume. The effect of trichomes on LAAPPI-MS analysis can be visually observed in the MS images, as discussed below.

Most metabolites were clearly detected across the frozen leaf, indicating good sensitivity of the LAAPPI-MS method. The lateral resolution of 70 μm allowed for imaging of leaf structures down to unicellular trichomes and tertiary veins, as discussed later in the imaging section. In addition, the use of frozen samples minimized the possible metabolic and other reactions during the measurements and allowed for the use of a relaxed dwell time between the measurements of each spot in imaging analysis. The main detected metabolites included different species of flavonol glycosides, fatty acids, esters, galactolipids, and glycosphingolipids. All these compounds are acidic and were detected as their deprotonated ions. The suggested ion species, accurate masses, and mass errors for the most abundant mass peaks detected from different leaf parts are presented in Table 1.

**Table 1:** Most Abundant Ions in the Different Parts of *A. thaliana* Leaves Detected with <10 ppm Mass Error^a^

part	molecular formula (tentative identification)	observed *m*/*z* [M – H]^−^	mass error (Δppm)	compound class	reference
trichome (body)	C_26_H_52_O_2_ (hexacosanoic acid)	395.387	4.8	saturated very-long-chain fatty acids	(30)
	C_28_H_56_O_2_ (octacontanoic acid)	423.417	7.6		
	C_30_H_60_O_2_ (triacontanoic acid)	451.449	5.5		
	C_32_H_64_O_2_ (dotriacontanoic acid)	479.480	5.9		
	C_34_H_68_O_2_ (tetratriacontanoic acid)	507.511	6.1		
	C_27_H_54_O_2_ (heptacosanoic acid)	409.402	6.2		
	C_29_H_58_O_2_ (nonacosanoic acid)	437.433	6.5		
	C_31_H_62_O_2_ (hentriacontanoic acid)	465.465	4.6		
	C_35_H_60_O_3_ (4-HCA hexacosyl ester)	527.444	4.6	alkyl hydroxycinnamates	
	C_37_H_64_O_3_ (4-HCA octacosyl ester)	555.473	8.5		
	C_39_H_68_O_3_ (4-HCA triacontyl ester)	583.509	6.9		
	C_41_H_72_O_3_ (4-HCA dotriacontanyl ester)	611.539	2.2		
	C_43_H_76_O_3_ (4-HCA tetratriacontyl ester)	639.567	7.2		
trichome (base)	C_21_H_20_O_10_ (K-Rha)	431.094	8.9	flavonol glycosides	(33−35)
	C_21_H_20_O_11_ (K-Glu or Q-Rha)	447.091	3.9		
	C_27_H_30_O_14_ (K-Rha-Rha)	577.152	6.5		
	C_27_H_30_O_15_ (K-Glu-Rha or Q-Rha-Rha)	593.147	6.2		
	C_28_H_32_O_16_ (I-Glu-Rha)	623.157	6.8		
	C_33_H_40_O_19_ (K-Glu-Rha-Rha)	739.204	6.2		
cuticle	C_30_H_54_O_3_ (18-(didecyloxy)-OA)	461.398	3.2	oxygenated fatty acid derivatives	(24 and 36)
	C_32_H_58_O_3_ (18-(tetradecyloxy)-OA)	489.426	9.8		
	C_32_H_58_O_4_	505.422	7.3		
	C_34_H_62_O_3_ (18-(hexadecyloxy)-OA)	517.457	9.9		
	C_34_H_62_O_4_	533.454	5.6		
	C_36_H_66_O_3_ (18-(octadecyloxy)-OA)	545.490	6.0		
	C_36_H_66_O_4_	561.485	5.9		
all veins	C_45_H_74_O_10_ (36:6-MDG)	773.518	3.1	galactolipids	(37)
	C_46_H_76_O_12_ (18:3/methyl-ketol-18:2-MGD)	819.518	9.6		
midvein	C_40_H_77_O_9_N (16:0-O/d18:1-GlcCer)	714.547	7.0	glucosylceramides	(38 and 39)
	C_40_H_77_O_10_N (16:0-O/t18:1-GlcCer)	730.542	6.7		
	C_48_H_91_O_10_N (t18:1/h24:1-GlcCer)	840.656	0.4		
tear and edge	C_55_H_74_N_4_O_5_ (pheophytin *a*)	869.552	7.0	chlorins	(40)
leaf lamina	C_28_H_32_O_14_ (1,2-disinapoylglucose)	591.168	5.7	sinapate esters	
	C_43_H_70_O_10_ (34:6-MGD)	745.486	4.1	galactolipids	(37)
	C_45_H_74_O_10_ (36:6-MGD)	773.518	3.1		
	C_44_H_72_O_12_ (18:3/methyl-ketol-16:2-MGD)	791.490	5.8		
	C_45_H_74_O_12_ (18:3/C18-ketol-MGD)	805.506	5.2		
	C_51_H_84_O_15_ (36:6-DGD)	935.567	6.6		

a K = kaempferol, Q = quercetin, I = isorhamnetin, Rha = rhamnoside, Glu = glucoside, OA = octadecatrienoic acid, 4-HCA = 4′-hydroxy-cinnamic acid, GlcCer = glucosylceramide.

### Trichomes

**Figure 3:**
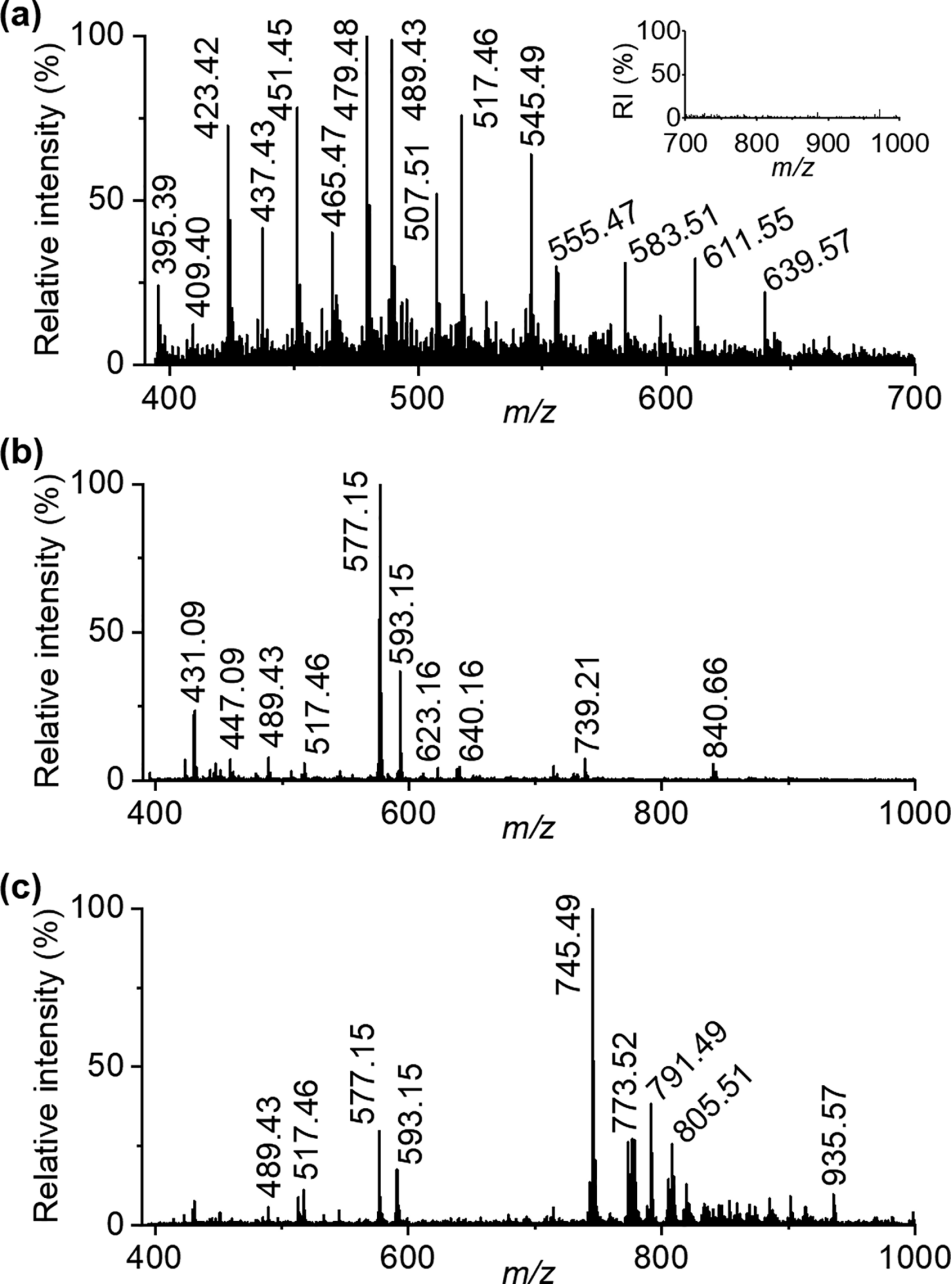
Example mass spectra acquired from (a) single-cell trichome, (b) trichome base, and (c) leaf lamina.

**Figure 4:**
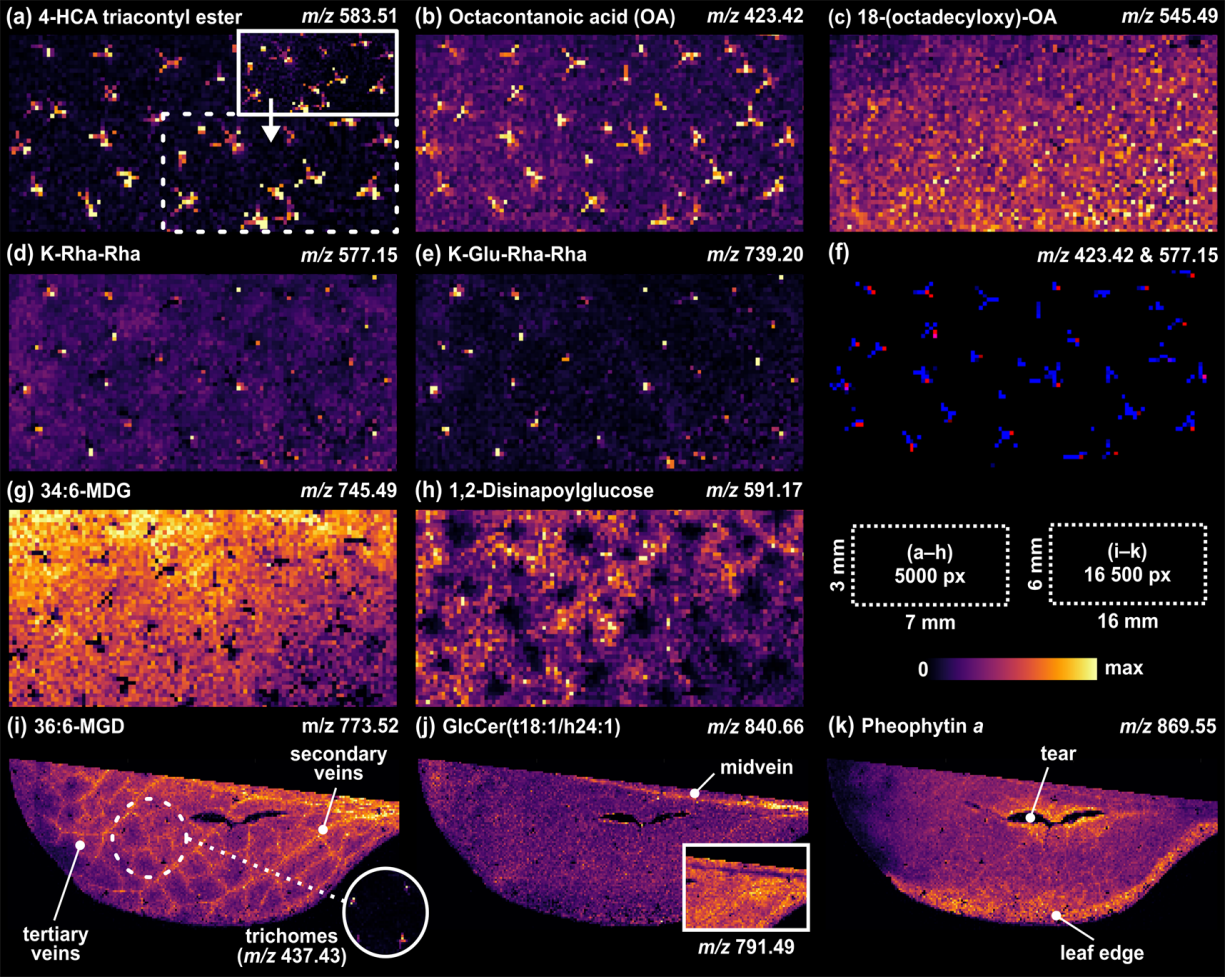
TIC-normalized LAAPPI-MS images of selected deprotonated ions acquired with 70 μm lateral resolution from frozen *A. thaliana* leaves. (a) Distribution of 4′-hydroxy-cinnamic acid (HCA) triacontyl ester revealing the star-shaped forms of single-cell trichomes with and without (top-right inset) normalization. (b) Distribution of octacontanoic acid. (c) Evenly distributed fatty acids of the uniform cuticular wax layer of the leaf. (d–f) Flavonol glycosides showing relatively higher abundances at the trichome base parts, as visualized with the colocalized image of (b) (blue) and (d) (red). (g) Evenly distributed galactolipid species showing the shadowing effect of trichome branches as black pixels. (h) Lack of 1,2-disinapoylglucose in the leaf tissue surrounding the trichome regions. (i and j) Lipid distributions showing the vein pattern or solely the midvein in one leaf half. (k) Pheophytin *a* had a relatively higher abundance in the surroundings of the tear and the edge of the leaf.

The unicellular trichomes of *A. thaliana* leaves are attached to the leaf lamina with basal cells, and they consist of stalks and typically 2–4 branches that point to different directions on the leaf surface (Figure 2).^19^ The length of the branches is a few hundred micrometers, and the diameter is approximately 10–20 μm, whereas the base of a trichome, consisting of stalk and basal cells, is approximately 100 μm in diameter. The profiles of the mass spectra acquired from the trichome regions varied pixel-by-pixel, showing a different number of compound classes depending on the number of consecutively sampled and analyzed leaf and trichome substructures (Figure 3). Negative ion LAAPPI-MS imaging also managed to spatially resolve single-cell trichomes from the *A. thaliana* leaf lamina and allowed for acquisition of images (Figure 4a,b) that clearly and repeatedly represent their star-shaped form. In addition, LAAPPI-MS imaging spatially resolved the base of a trichome from the rest of its body. Due to the used TIC normalization and the effect of trichomes on sampling, the ions listed were also ensured to have the highest detected absolute intensity at the presented leaf parts in Table 1 and Figure 4.

#### Single-Cell Trichome

The main compounds having clearly higher abundances in the unicellular trichomes than in the other parts of the leaf are fatty acids and their esters. The mass spectrum detected from one trichome branch shows three different trichome-specific homologous series of peaks with a mass difference of 28 amu (Figure 3a, Table 1), indicating that the compounds include a long alkyl chain. On the basis of the accurate masses, the first two ion series at the mass ranges of *m*/*z* 395–507 (C_26_–C_34_) and *m*/*z* 409–465 (C_27_–C_31_) consist of deprotonated even and odd chain saturated very-long-chain fatty acids (VLCFAs), respectively. This result is consistent with a previous study by Ebert and co-workers,^30^ who identified VLCFAs from harvested *A. thaliana* leaf trichomes by gas chromatography–mass spectrometry (GC–MS). The members of the third homologous series at the mass range of *m*/*z* 527–639 (Figure 3a) are well-ionized in the negative ion mode, which suggests that they include an acidic group. On the basis of the accurate masses, the members of the third homologous series match 4′-hydroxycinnamic acid (4-HCA) esters, which belong to a class of alkyl hydroxycinnamates (ACHs). ACHs are natural products in various plant tissues and have been reported in more than 50 plant species.^31^ The LAAPPI-MS images of the detected saturated fatty acids and 4-HCA esters (Table 1), octacontanoic acid (*m*/*z* 423.42) and 4-HCA triacontyl ester (*m*/*z* 583.51), as examples, show that their ion distributions correlate well with the branched shape of the single-cell trichomes. The intensities of the reported fatty acids were significantly higher in the single-cell trichomes than in the trichome bases or leaf lamina (Figure 4a,b), and 4-HCA esters were detected only in the trichomes. In addition, the mass spectrum measured from the trichome branch (Figure 3a) shows the fourth homologous series, which is not specific to the trichome but to the leaf cuticle (see the discussion below).

#### Trichome Base

The mass spectra measured from the base parts of the trichomes (Figure 3b) are clearly different than those measured from the rest of the trichome body (Figure 3a) or the leaf lamina (Figure 3c). The accurate masses of the most intense peaks indicate that they are deprotonated flavonol glycosides, including species such as kaempferol (K), quercetin (Q), or isorhamnetin(I), with sugars such as rhamnose (Rha) and glucose (Glu) (Table 1), which are well-known and one of the most abundant secondary metabolite groups in *A. thaliana* leaves and trichomes.^32,33^ The LAAPPI-MS images (Figure 4d,e) of the flavonol glycosides, K-Rha-Rha (*m*/*z* 577.15) and K-Glu-Rha-Rha (*m*/*z* 739.20), as examples, reveal a clearly higher intensity for these species at small specific leaf areas consisting of only a few pixels. The image (Figure 4f) containing colocalized distributions of K-Rha-Rha and octacontanoic acid (*m*/*z* 423.42), which is specific for the whole trichome (see above), confirms that flavonol-rich pixels (red) are located at the base parts of the trichomes, either in the lower part of the trichome stalk or in the basal cells that surround the stalk and attach the trichome to the leaf surface (Figure 2b). Some of the flavonol glycoside species detected have also previously been reported to be more abundant at the base parts than in the leaf lamina by a microsampling-based ESI-MS method,^34^ supporting our LAAPPI-MS data. It has been shown that zinc accumulates at the base of trichomes of *A. thaliana* leaves,^17^ and as zinc can form chelates with flavonoids,^41^ this may partly explain why flavonol glycosides were significantly more abundant at the base parts of the trichomes than in the other parts of the leaf.

#### Leaf Lamina

The mass spectrum acquired from the leaf lamina region differs from that of the trichome and its base part by containing many intense lipid peaks in the mass range *m*/*z* 700–1000 (Figure 3c). The accurate masses of these lipids (Table 1) match the oxidized and nonoxidized galactolipids of *A. thaliana* leaves detected in earlier studies^37,42^ and include many monogalactosyldiacylglycerol (MGD) and digalactosyldiacylglycerol (DGD) species, such as 36:6-MGD (*m*/*z* 773.52) and 34:3-DGD (*m*/*z* 913.59). MGD and DGD species alone compose approximately 60% of all leaf lipids or 80% of membrane lipids in chloroplasts within the mesophyll cells below the epidermis (Figure 2b).^42^ Most of the detected galactolipids were distributed quite evenly across the leaf (Figure 4g), with 34:6-MGD (*m*/*z* 745.49) as an example. Figure 4 also shows that the intensity of 34:6-MGD at the some trichome pixels is low compared to that in the other leaf lamina regions, which may be caused by the interaction of the trichome branches with the IRLA sampling and thus the analysis of leaf layers under the branches, as discussed above. This result also shows that 34:6-MGD was not detected in the measured branches. The mass spectrum (Figure 3c) acquired from the leaf lamina also shows several mass peaks, such as flavonol glycosides and saturated fatty acids, which had a higher abundance in the mass spectra of the trichome body and its base. On the contrary, the MS image of *m*/*z* 591.17 (Figure 4h) shows that this ion has a significantly lower abundance at the trichome and around its proximity than at the other regions of the leaf. The accurate mass of this ion matches that of 1,2-disinapoylglucose (1,2-DSG), which is a common secondary metabolite in leaves but not in trichomes.^33,43^ The abundance of the ion at *m*/*z* 869.55 was significantly higher at the leaf edge than at the other parts of the leaf lamina (Figure 4k). The same was observed in the surroundings of the tear, which was obviously formed in the sample preparation process. The accurate mass of the ion matches with pheophytin *a*, which is a chlorophyll *a* molecule without Mg^2+^ ion. Pheophytin *a* acts as an electron carrier in photosynthesis, and it can be formed due to leaf senescence or a metabolic stress reaction in the tissue.^44,45^ It is unclear, however, whether the molecule in the sample is pheophytin *a* or chlorophyll *a*, which can be fragmented to pheophytin *a* by loss of Mg in the ion source, as also observed in the earlier LAESI study.^46^

### Veins

LAAPPI-MS images of half of a frozen *A. thaliana* leaf revealed vein patterns (Figure 4i,j), including a midvein, secondary veins, and high-order tertiary veins whose diameters were only tens of μm.^47^ The mass peaks having a higher intensity in the veins than in the other parts of the leaf are all lipids detected in the mass range *m*/*z* 700–1000 (Table 1). On the basis of the accurate masses and the known lipid composition of *A. thaliana* leaves,^37,38^ the most abundant lipids in veins are galactolipids and glucosylceramides (GlcCer) (Table 1). The identification of GlcCer lipids is also supported by the similar distributions of ceramide fragment ions, such as Cer(t18:1/h24:1) detected at *m*/*z* 678.60 (data not shown). All the detected GlcCer lipids (Table 1) have also been previously detected in *A. thaliana* leaves by LC–MS.^39^ The distribution of galactolipids, galactolipid 36:6-MDG (*m*/*z* 773.52) as an example (Figure 4i), clearly correlates with all the vein structures in the leaves (Figure 4i). As an exception, glucosylceramides (GlcCer), GlcCer (t18:1/h24:1) (*m*/*z* 840.66) as an example (Figure 4j), were most abundant only in the midvein (Table 1). Additionally, some galactolipids were less abundant in the midvein, as shown in the image (Figure 4j inset) of 18:3/methyl-ketol-16:2-MGD (or methyl-ketol-18:2/16:3-MGD, *m*/*z* 791.49).

### Depth Profiling

**Figure 5:**
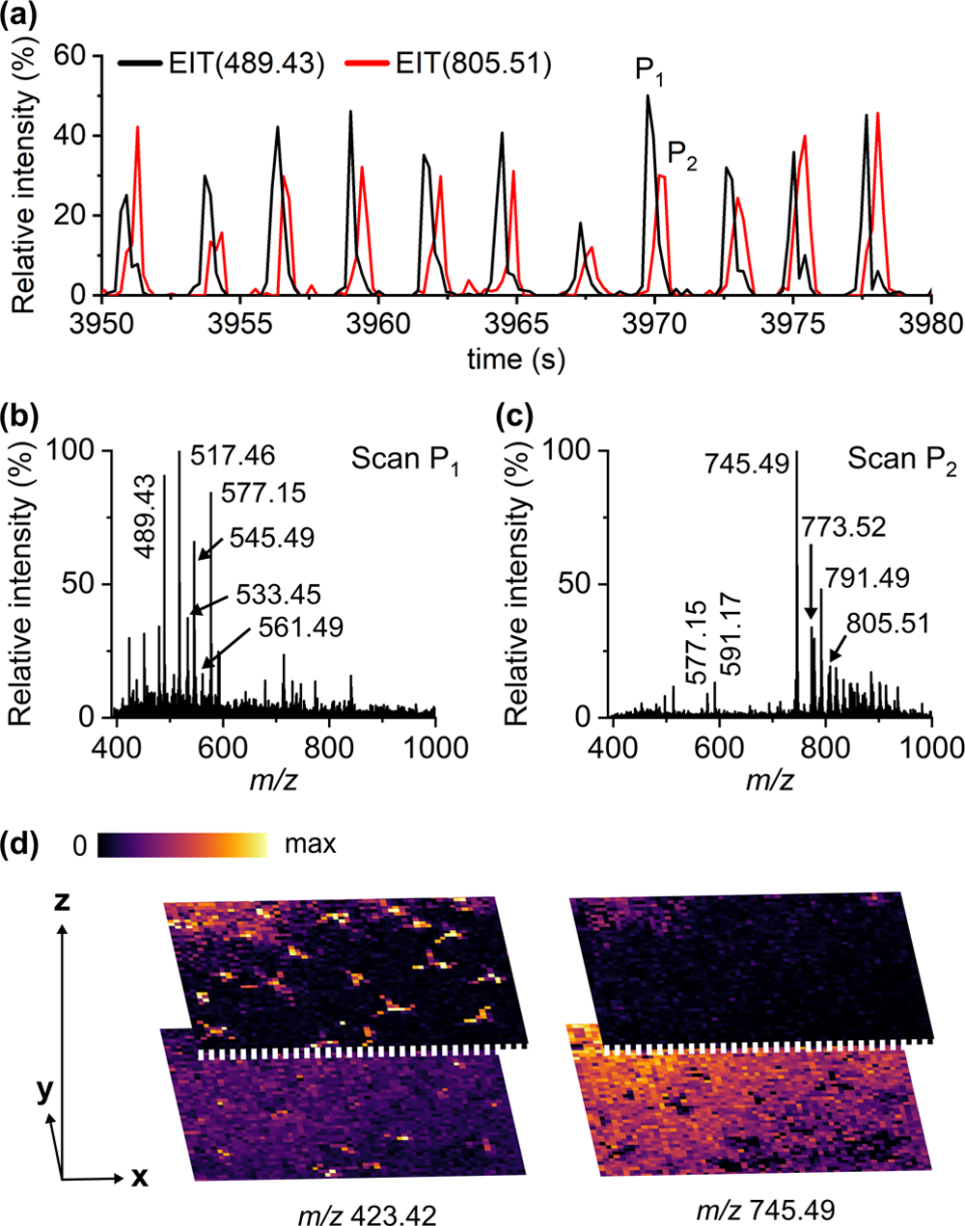
Depth profiling of the leaf lamina by LAAPPI-MS. (a) Extracted ion traces (EIT) of 18-(tetradecyloxy)-octadecatrienoic acid (*m*/*z* 489.43) and 18:3/C18-ketol-MGD (*m*/*z* 805.51) measured at different times from one 65 μm diameter spot. The peak pairs (e.g., P_1_ and P_2_) of each spot were separated by 2–3 scans with a data acquisition rate of 5 Hz. (b and c) Extracted mass spectra of peak P_1_ and P_2_ scans. (d) Distributions of octacontanoic acid (*m*/*z* 423.42) and 34:6-MGD (*m*/*z* 745.49) above and below the leaf surface.

*A. thaliana* leaves have a complex, multilayered structure (Figure 2b) of approximately 150 μm in thickness,^48^ which provides an excellent model for examining the feasibility of the pulsed (20 Hz) IR laser ablation sampling-based LAAPPI-MS for depth profiling. As the emitted IR pulses reach the leaf surface, they begin to ablate the lamina pulse-by-pulse, which allows different leaf layers of each measurement spot to be sampled and analyzed at different times. The extracted ion traces (EIT) generated from the LAAPPI-MS imaging data of the leaf lamina show that some of the compounds were repeatedly detected at different times (Figure 5a) during the analysis of each spot (EIC peak). This indicates that LAAPPI-MS could spatially resolve at least two structures located at different depths of the leaf. Although the EIT peaks are not completely separated with an acquisition rate of 5 Hz, there were clear differences in the mass spectral profiles of the peak scans P_1_ and P_2_ (Figure 5b,c), which represent the data acquired from the upper and deeper parts of the leaf, respectively. The mass spectrum of the peak scan P_1_ (Figure 5b) shows two homologous ion series at the mass ranges *m*/*z* 461–545 and *m*/*z* 505–561. The accurate masses indicate that the ion series consists of oxygenated (C_30_–C_36_) fatty acid derivatives (Table 1), some of which have been detected in earlier studies by GC–MS^36^ and MALDI-MSI^24^ from the topmost cuticle layer of the leaf (Figure 2b). The oxygenated fatty acid derivatives, 18-(octadecyloxy)-octadecatrienoic acid (*m*/*z* 545.49) as an example (Figure 4c), were evenly distributed on the *A. thaliana* leaf, as the cuticle covers the entire leaf surface, including the trichomes. The mass spectrum of peak scan P_1_ also contains flavonol glycosides, which were found most abundant at the trichome bases, as discussed above. Flavonol glycosides are known to protect plants from UV radiation, which explains their detection from the topmost parts of the leaf (epidermis). The mass spectrum of peak scan P_2_ (Figure 5c) shows many lipid peaks at the mass range of *m*/*z* 700–1000 and has a similar profile as the mass spectrum acquired from the interveinal parts of the leaf lamina (Figure 3c). As discussed above, most of these lipid peaks belong to different galactolipid species that are most abundant in mesophyll layers below the epidermis.

The feasibility of LAAPPI-MS for the imaging of leaf parts at different depths (*z*-axis) can be demonstrated by halving the MS data of each measured spot to sets that represent the top and bottom parts of the leaf (Figure 5d). The data presented covers approximately 300 μm in depth as the trichomes expand above the leaf surface roughly the same distance as the thickness of the leaf (150 μm, Figure 2b). The differences in the analyte depth distributions are observed most clearly with the species that are rich in the surface trichomes and the subepidermal mesophyll cells. The example MS images of the top parts of the leaf show that the intensity of octacontanoic acid (*m*/*z* 423.42) is clearly the highest at the location of the trichomes as its distribution clearly reveals their star-shaped form. Otherwise, the intensity of octacontanoic acid is lower than that it is in the bottom half of the leaf, in which the distribution of this species is more uniform as there are no trichomes below the leaf surface. In the other example, the intensity of a galactolipid 34:6-MGD (*m*/*z* 745.49) is low in the top parts and high in the bottom parts of the leaf due to the high abundance of galactolipids below the epidermis. Similar depth distributions were detected for pheophytin *a* (data not shown) that is most abundant in the chloroplasts below the epidermis. Overall, the results indicate that LAAPPI-MS is capable for profiling and MS imaging of structures that are located either above or at different depths of *A. thaliana* leaves, which suggests that the method could be developed further for three-dimensional (3-D) MS imaging without sample sectioning, as previously shown by other IR laser ablation-based methods such as LAESI.^49,50^

### Role of Metabolites

Leaf trichomes have been reported to protect plants mechanically from herbivorous insects and defend against harmful UV radiation and excess light.^19^ The composition of the *A. thaliana* trichome waxes (e.g., hydrocarbons, alcohols, ketones, esters, fatty acids, etc.) has been reported to differ from the wax composition of the rest of the leaf by favoring very-long-chain hydrocarbon biosynthesis with chain lengths longer than C_32_,^51^ matching the esters detected solely from the trichomes by LAAPPI-MS. The high abundance of VLCFAs may influence the melting behavior of the cuticular wax and the mechanical properties of the trichomes by affecting their flexibility or rigidity.^51^ Moreover, high concentrations of VLCFAs and alkyl hydroxycinnamates may reduce the evaporation of water through the cuticle, which is particularly important for trichomes, as they have a much higher surface area per volume than the other epidermal cells.^51^ The alkyl hydroxycinnamates are also likely to contribute to UV screening together with the flavonol glycosides of the trichome basal parts.

Although glycosylated inositol phosphorylceramides are the most abundant class of sphingolipids in *A. thaliana*,^52^ negative ion LAAPPI-MS detected various glucosylceramides that were found most abundant in the midvein. Different plant tissues have been shown to vary their sphingolipid composition, but little is known about the functional significance of the varying structures. Subepidermal mesophyll cells contain the majority of leaf chloroplasts that have membranes highly enriched in octadecatrienoic (C_18_) acid-containing galactolipids.^53^ On the contrary, discrimination of cuticular fatty acid esters from chloroplastic galactolipids in the interveinal areas of the leaf lamina demonstrated the promise of the approach for depth profiling analysis.

## Conclusions

OPO IR laser beams can now be focused on spot sizes of tens of micrometers, which significantly improves the applicability of MS imaging methods on the basis of IR laser ablation sampling. LAAPPI-MS imaging with 70 μm lateral resolution was capable of showing different distributions of metabolites at different leaf parts, such as veins, cuticle, and even single-cell trichomes, which has not been previously shown by ambient MS imaging. The different species of fatty acids and their esters showed distributions that matched well with the star-shaped form of a single-cell trichome. The basal cells or the stalk of the trichome were also spatially resolved from the trichome body by their relatively higher flavonol glycoside concentration. In lipid imaging, galactolipids were found to be most abundant in the interveinal leaf lamina, and some of their distributions revealed the leaf vein pattern. Different species of glucosylceramides and their distributions revealed solely the midvein instead. In addition, LAAPPI-MS showed its potential for depth profiling analysis for the first time by mapping analytes at the different levels of the leaf tissue and spatially resolving the topmost trichomes and cuticle layer from the underlying parts of the leaf. The results show that LAAPPI-MS with sub-100 μm lateral resolution offers a highly promising method to study the effects of biotic and abiotic factors on the abundances of metabolites in different substructures of the leaves, providing detailed information on the roles of metabolites in plants.
